# Apoptosis of THP-1 Derived Macrophages Induced by Sonodynamic Therapy Using a New Sonosensitizer Hydroxyl Acetylated Curcumin

**DOI:** 10.1371/journal.pone.0093133

**Published:** 2014-03-27

**Authors:** Longbin Zheng, Xinyong Sun, Xing Zhu, Fengxiang Lv, Zhaoyu Zhong, Feng Zhang, Wenhui Guo, Wenwu Cao, Liming Yang, Ye Tian

**Affiliations:** 1 Department of Pathophysiology, The Key Laboratory of Myocardial Ischemia, Ministry of Education, Harbin Medical University, Harbin, Heilongjiang, China; 2 Division of Cardiology, The First Affiliated Hospital, Harbin Medical University, Harbin, Heilongjiang, China; 3 Institute of Molecular Medicine, State Key Laboratory of Biomembrane and Membrane Biotechnology, Peking University, Beijing, China; 4 Laboratory of Sono- and Photo-Theranostic Technologies, Harbin Institute of Technology, Harbin, Heilongjiang, China; 5 Materials Research Institute, Pennsylvania State University, University Park, Pennsylvania, United States of America; Cardiological Center Monzino, Italy

## Abstract

Curcumin is extracted from the rhizomes of the traditional Chinese herb *Curcuma longa*. Our previous study indicated curcumin was able to function as a sonosensitizer. Hydroxyl acylated curcumin was synthesized from curcumin to eliminate the unstable hydroxy perssad in our group. The potential use of Hydroxyl acylated curcumin as a sonosensitizer for sonodynamic therapy (SDT) requires further exploration. This study investigated the sonodynamic effect of Hydroxyl acylated curcumin on THP-1 macrophage. THP-1 macrophages were cultured with Hydroxyl acylated curcumin at a concentration of 5.0 μg/mL for 4 hours and then exposed to pulse ultrasound irradiation (0.5 W/cm^2^ with 1.0 MHz ) for 5 min, 10 min and 15 min. Six hours later, cell viability decreased significantly by CCK-8 assay. After ultrasound irradiation, the ratio of apoptosis and necrosis in SDT group was higher than that in control, Hydroxyl acylated curcumin alone and ultrasound alone. Moreover, the apoptotic rate was higher than necrotic rate with the flow cytometry analysis. Furthermore, Hydroxyl acylated curcumin-SDT induced reactive oxygen species (ROS) generation in THP-1 macrophages immediately after the ultrasound treatment while ROS generation was reduced significantly with the scavenger of singlet oxygen Sodium azide (NaN_3_). Hydroxyl acylated curcumin-SDT led to a conspicuous loss of mitochondrial membrane potential (MMP) compared with other groups, while MMP was increased significantly with the scavenger of singlet oxygen Sodium azide (NaN_3_), ROS inhibitor N-acetyl cysteine (NAC) and Mitochondrial Permeability Transition Pore (MPTP) inhibitor Cyclosporin A (CsA). The cytochrome C, cleaved-Caspase-9, cleaved-Caspase-3 and cleaved-PARP upregulated after SDT through Western blotting. These findings suggested that Hydroxyl acylated curcumin under low-intensity ultrasound had sonodynamic effect on THP-1 macrophages via generation of intracellular singlet oxygen and mitochondria-caspase signaling pathway, indicating that Hydroxyl acylated curcumin could be used as a novel sonosensitizer in SDT for atherosclerosis.

## Introduction

Atherosclerosis is one of the most common chronic inflammatory vascular diseases in clinical patients, which poses a severe threat to human health [1]. Studies showed that the sudden rupture of atherosclerosis was the crucial point of most acute cardiovascular events [2]. Macrophage is one of the most important inflammatory cells in unstable atherosclerosis plaque, which plays an indispensable role at all stages of atherosclerosis [3]–[4]. Therefore, the removal of macrophages from unstable plaque displays an effective strategy for atherosclerosis [5]–[6].

Our group proved that 5-Aminolevulinic acid (ALA) mediated photodynamic therapy (PDT) could reduce macrophage content and inhibit plaque progression in rabbit carotid artery atherosclerosis models [7]–[8] **.** However, the application of PDT was limited to superficial lesions because of light definite penetration. Unlike PDT, sonodynamic therapy (SDT) could penetrate deeply into tissues, which is one promising treatment for tumors *in vitro* and *in vivo* [9]–[11]. Sonication could minimize the damage to surrounding normal tissues for localization to pathological target cells [12]–[14]. The generation of reactive oxygen species (ROS) from the ultrasonic-activated sonosensitizer leads to an attack on the cell mitochondria, which is responsible for the sonodynamic damage [15]–[16]. Most sonosensitizers come from photosensitizers which make them liable to cause photodermatitis and they are not generally used in clinical practice [17]–[18]. It is indispensable to develop a widely used sonosensitizer for avoiding photodermatitis. Recently our group indicated that both emodin and curcumin had a sonodynamic effect on macrophages [19]–[20]. Emodin and curcumin mediated SDT decreased macrophages viability obviously and induced apoptosis of macrophages *in vitro*.

Curcumin is extracted from the rhizomes of the traditional Chinese herb *Curcuma longa* L (Zingiberaceae), which has antiinflammatory, antimutagenic and anticarcinogenic activities [21]. Moreover, our group has proved that curcumin could be used as a sonosensitizer [20]. Since hydroxy of curcumin is an unstable perssad, which decomposes easily, we acetylated the hydroxy of curcumin, named Hydroxyl acylated curcumin (HAC). But it is unknown that whether HAC-SDT is able to induce macrophage apoptosis. In this study we evaluated the effect of combining a much lower concentration of HAC with low-intensity ultrasound. The purpose was to verify if low-intensity ultrasound could enhance the macrophage apoptosis at a low concentration of HAC and to identify the possible mechanism responsible for the effects of HAC-SDT on THP-1 macrophages *in vitro*.

## Materials and Methods

### Ultrasonic exposure system

**Figure 1:**
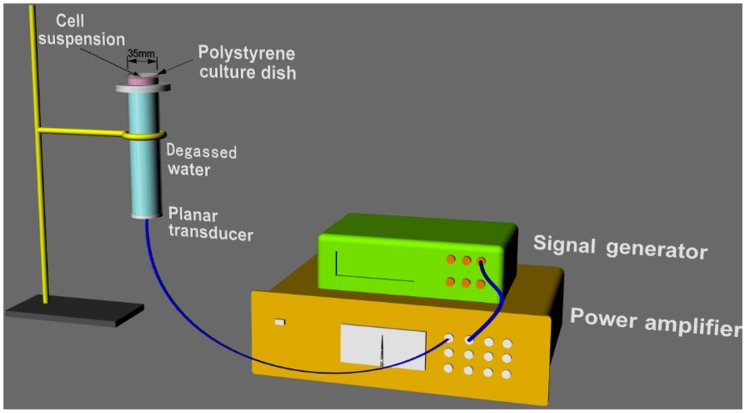
Schematic diagram of sonication devices for the *in vitro* experiments. The tone-burst ultrasonic transducer (1.0 MHz center frequency, 10% duty factor) was fixed by aluminum stents facing upward. The culture dish was placed above the center of the transducer for the *in vitro* experiments. The transducer was placed in a degassed water bath 30 cm under the cells.

The ultrasonic generator and power amplifier (Fig. 1) developed by the Condensed Matter Science and Technology Insitute, Harbin Institute of Technology (Harbin, China). The transducer (diameter 3.5 cm, resonance frequency 1.0 MHz, duty factor 10%, repetition frequency 100 Hz) was placed in the degassed water bath and the cells were placed 30 cm away from the transducer. The ultrasonic intensity was 0.5 W/cm^2^ measured using a hydrophone (Onda Corporation, Sunnyvale, CA, USA). During the sonication procedure, the temperature of the solution inside the Petri dishes increased less than 0.5°C, measured using a thermometer.

### Cell culture

Human THP-1 cells (American Type Culture Collection, Manassas, VA, USA) were seeded at a density of 1.0×10^5^ cells per milliliter in RPMI 1640 medium (HyClone, Logan, UT, USA) containing 10% FBS (HyClone, Logan, UT, USA), 20 μg/mL penicillin and 20 μg/mL streptomycin (St Louis, MO, USA). The cells were maintained at 37°C in a humidified atmosphere containing 5% CO_2_. The cells were differentiated into macrophages by adding 100 ng/mL Phorbol-12-myristate-13-acetate (PMA) (La Jolla, CA, USA) for 72 hours in 96-well plates, 24-well plates and 35 mm Petri dishes.

### The synthesis of Hydroxyl acylated curcumin

To a well-stirred of curcumin (1.0 mmol, 386 mg) in anhydrous ACOOEt (20 mL) was added Acetic (3.0 mmol, 255 mg) and H_2_SO_4_ (0.05 mL).The reaction mixture was at 60°C for 1 h. After the starting material curcumin was consumed as indicated by TLC, the resulting mixture was allowed to cool down to room temperature, the reaction mixture was poured into water (10 mL) under stirring. Then the reaction mixture was extracted with ACOOEt (3×30 mL). The combined organic phase was washed with water (3×20 mL), dried over MgSO_4_, filtered and concentrated in vacuo. The crude product was recrystallized from methanol/acetonitrile to give acylated curcumin (370 mg, 95%) as a yellow solid.

### Intracellular accumulation of Hydroxyl acylated curcumin

The cells were incubated with 5.0 μg/mL HAC for 15 min, 30 min, 1 h, 2 h, 3 h, 4 h, 5 h, 6 h, 7 h, 8 h. Intracellular HAC (excitation wavelength 407 nm, emission wavelength 476 nm) was identified using a laser confocal microscopy (LSM 510 Meta; Zeiss, Gottingen, Germany). The fluorescence intensity of HAC was processed and analyzed with the Zeiss CLSM software (ZEN 2009 Light Edition).

### Cell viability assay

The cells were seeded into 96-well plates and incubated with HAC for 4 hours. The survival rates of the cells were measured by colorimetric assay with CCK-8 (Beyotime Biotech, China). During the experiment, the cells were seeded into the 96-well plate and incubated at 5.0 μg/mL HAC for 4 hours in the dark, then exposed to the ultrasound for 5, 10 and 15 minutes. After 6 hours following HAC-SDT treatment, the survival rates of the cells were measured by CCK-8 assay. Experiments were repeated at least three times independently.

### Flow cytometry analysis

Cell apoptosis and necrosis were assessed by the Annexin V-FITC apoptosis kit (Franklin Lakes, NJ, USA) according to the manufacturer’s instructions. Cells were divided randomly into four groups (control, ultrasound alone, HAC alone and SDT). For the HAC alone and SDT groups, 5.0 μg/mL HAC solution was added to the cells. After 4-hour incubation in the dark, the cells in the ultrasound alone and SDT groups were exposed to the ultrasound for 5 minutes. After 6 hours, the cells were incubated with 5 μL Annexin V and 5 μL PI for 10 minutes at room temperature in the dark. Cells from each sample were then analyzed by FacsCalibur flow cytometer (BD Biosciences). The data were analyzed using CELLQuest software (BD). The results were interpreted in the following fashion: cells in the lower-left quadrant (Annexin-V^−^/PI^−^) represent living cells; those in the lower-right quadrant (Annexin-V^+^/PI^−^) represent early apoptotic cells; those in the upper-right quadrant (Annexin-V^+^/PI^+^) represent late apoptotic cells; those in the upper-left quadrant (Annexin-V^−^/PI^+^) represent necrotic cells. Experiments were repeated at least three times independently.

### Mitochondrial membrane potential detection

Mitochondrial membrane potential (MMP) was assessed by fluorescent probe jc-1 (Beyotime Biotech, China). After 6 hours following HAC-SDT treatment, macrophages were incubated with 10 mg/mL jc-1 for 20 minutes at 37°C in the dark and were monitored by the laser confocal microscopy and fluorescence microscope. Red-orange fluorescence was attributable to a potential-dependent aggregation in the mitochondria. Green fluorescence, reflecting the monomeric form of jc-1, appeared in the cytosol after mitochondrial membrane depolarization. The fluorescence intensity was measured with a fluorospectrophotometer (Varian Australia Pty Ltd, Melbourne, Victoria, Australia) at 488 nm excitation, 530 nm (green) and 590 nm (red) emission wavelengths. In experiments involving the scavenger of singlet oxygen Sodium azide (NaN_3_), ROS inhibitor N-acetyl cysteine (NAC) and Mitochondrial Permeability Transition Pore (MPTP) inhibitor Cyclosporin A (CsA), the cells were pre-treated with 10 mM NaN_3_, 1.0 mM NAC or 0.5 μM CsA, immediately followed by SDT. Experiments were repeated at least three times independently.

### ROS measurement

Intracellular ROS content was determined by measuring the fluorescence of 2′,7′-dichlorofluorescein (DCF). 2′,7′-dichlorofluorescein diacetate (DCFH-DA) (Applygen Technologies Co. Ltd., Beijing, China) was added to the medium of cells at a final 20 μM concentration and incubated at 37°C for 30 minutes. The cells were then washed with PBS twice carefully. Immediately after HAC-SDT treatment, measured using the fluorospectrophotometer at 488 nm excitation and 525 nm emission wavelengths. In experiments concerning the singlet oxygen scavenger, sodium NaN_3_, the cells were pretreated with 10 mM NaN_3_ before SDT. The scavenger at the concentrations used did not cause any significant damage in the cultured cells. Experiments were repeated at least three times independently.

### Western blot

SDS-PAGE and immunoblotting were performed according to standard procedures to assay the expression levels of apoptosis-associated proteins including cytochrome C, cleaved-Caspase-9, cleaved-Caspase-3 and cleaved-PARP. Briefly, after 6 h incubation following SDT treatment, cells were harvested and collected by centrifugation, and then lysed with RIPA buffer (containing 50 mM Tris-HCl (pH 7.4), 150 mM NaCl, 1 mM EDTA, 1% Triton X-100, 1% sodium deoxycholate, 0.1% Sodium dodecyl sulfate (SDS), 1 mM Phenylmethanesulfonyl fluoride (PMSF), 1 μM leupeptin and 0.01 μM aprotinin). The protein content of the lysate was measured using the Bicinchoninic acid (BCA) protein assay reagent. Similar amount of protein was analyzed on sodium dodecyl sulfate-polyacrylamide gel electrophoresis gel (SDS-PAGE) and transferred onto polyvinylidene fluoride membranes. Membranes were then incubated at the room temperature for 1 h in blocking buffer (5% low-fat milk powder in tris-buffered saline-tween 20 (0.05%) (TBST). The membranes were incubated overnight at 4°C with primary antibodies (cytochrome C, cleaved-Caspase-9, cleaved-Caspase-3 and cleaved-PARP were from Cell Signaling Technology, Inc, USA). All antibodies dilutions were 1∶1000. The bound primary antibodies were then tagged with horseradish peroxidase-labeled secondary antibodies at the room temperature for 1 h, and the immune complexes were detected by enhanced chemiluminescence reagents. Anti-β-actin (Cell Signaling Technology, Inc, USA) was used to ensure equal loading. Experiments were repeated at least three times independently.

### Statistical analysis

All experiments were independently performed at least 3 times. Data were analyzed using one-way ANOVA and presented as mean ± standard deviation (SD) values. Values that reached a p<0.05 level of significance were considered statistically significant.

## Results

### Physical optics characterization of HAC

**Figure 2:**
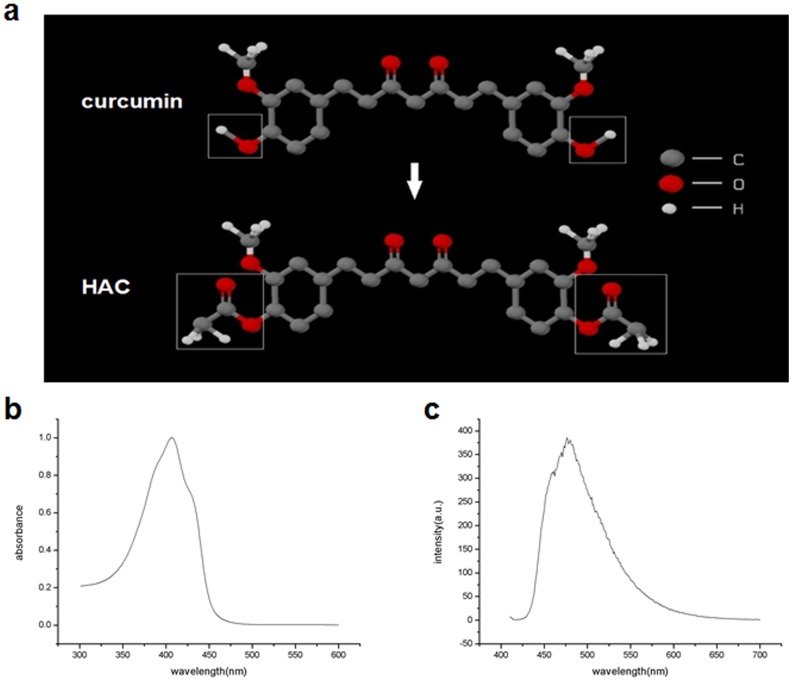
The structure and physical optics characterization of Hydroxyl acetylated curcumin. (a) The structure of Hydroxyl acetylated curcumin (HAC). (b) The absorption spectrum of HAC. (c) The fluorescence emission spectrum of HAC.

The structure of HAC was shown in Fig. 2a, the excitation wavelength of HAC was 407 nm, and the fluorescence emission wavelengths of HAC ranged from 450 nm to 600 nm (Fig. 2b, c).

### Cell viability

**Figure 3:**
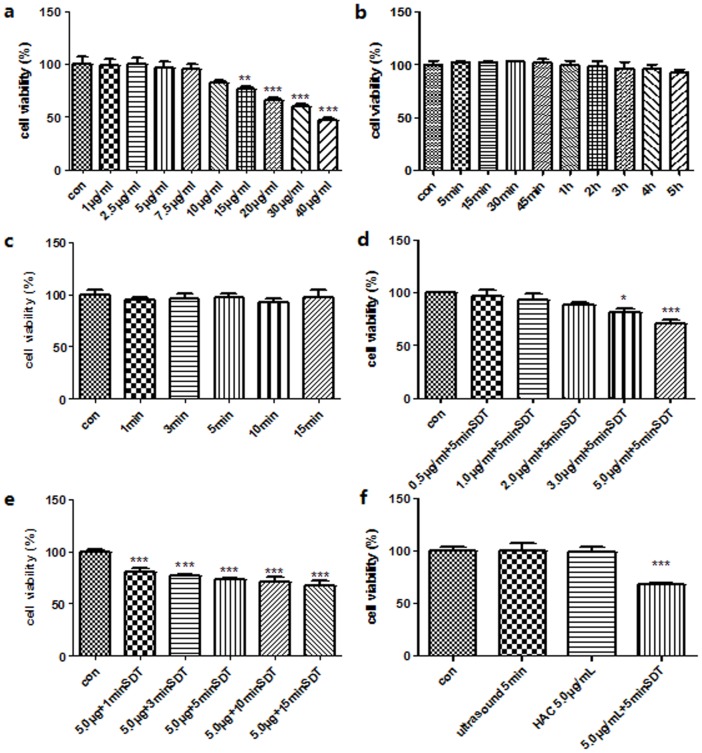
The survival rates of THP-1 macrophages determined by CCK-8 assay. (a) The survival rates of THP-1 macrophages after different concentration of HAC incubation for 4 h. (b) The survival rates of THP-1 macrophages after 5.0 μg/mL HAC incubation with different time. (c) The survival rates of THP-1 macrophages after ultrasound exposure for 0, 1, 3, 5, 10 and 15 minutes. (d) The survival rates of THP-1 macrophages after 5 minutes ultrasound exposure with different HAC concentrations. (e) The survival rates of THP-1 macrophages after 0, 1, 3, 5, 10 and 15 minutes ultrasound exposure with 5.0 μg/mL HAC concentrations. (f) The survival rates of THP-1 macrophages after different treatment, control, ultrasound alone, 5.0 μg/mL HAC alone and SDT (5.0 μg/mL HAC plus 5 minutes ultrasound exposure). *p<0.05 **p<0.01 ***p<0.001 *vs.* control.

The CCK-8 assay showed that the survival rates of THP-1 macrophages decreased with different HAC concentration and ultrasound exposure time. The survival rate decreased significantly at 15 μg/mL HAC concentration (Fig. 3a; p<0.01). 5.0 μg/mL HAC concentration had no effect on the cell viability (Fig. 3b). The survival rates had no influence with ultrasound exposure alone for 1, 3, 5, 10 and 15 minutes (Fig. 3c). The survival rate decreased significantly in cells treated with 3.0 μg/mL and 5.0 μg/mL HAC concentration, using 5 minutes ultrasonic irradiation compared with control (Fig. 3d; p<0.05). The survival rates decreased significantly in cells treated with 5.0 μg/mL HAC concentration under different ultrasonic irradiation time (Fig. 3e; p<0.05). According to optimized conditions (5.0 μg/mL HAC, 5 minutes ultrasound exposure), the survival rate decreased significantly in SDT group (Fig. 3f; p<0.01).

### Intracellular accumulation of Hydroxyl acylated curcumin

**Figure 4:**
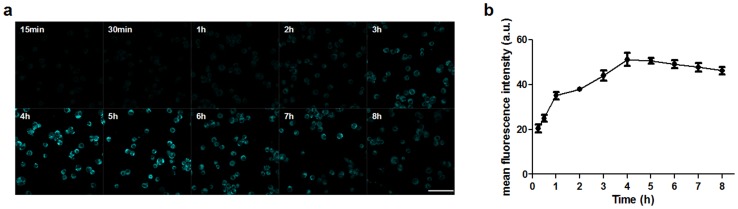
Intracellular Accumulation of Hydroxyl acetylated curcumin detected by laser confocal microscopy. (a) Fluorescent photomicrograph of HAC in THP-1 macrophages at different times after incubation with 5.0 μg/mL HAC. (b) The accumulation of HAC in THP-1 macrophages. Scale bar  =  0.05 mm.

The fluorescence intensity of Hydroxyl acylated curcumin by the laser confocal microscopy increased in THP-1 macrophages (Fig. 4a), peaked at 4 hours, and then faded with time (Fig. 4b).

### Apoptosis and Necrosis of cells

**Figure 5:**
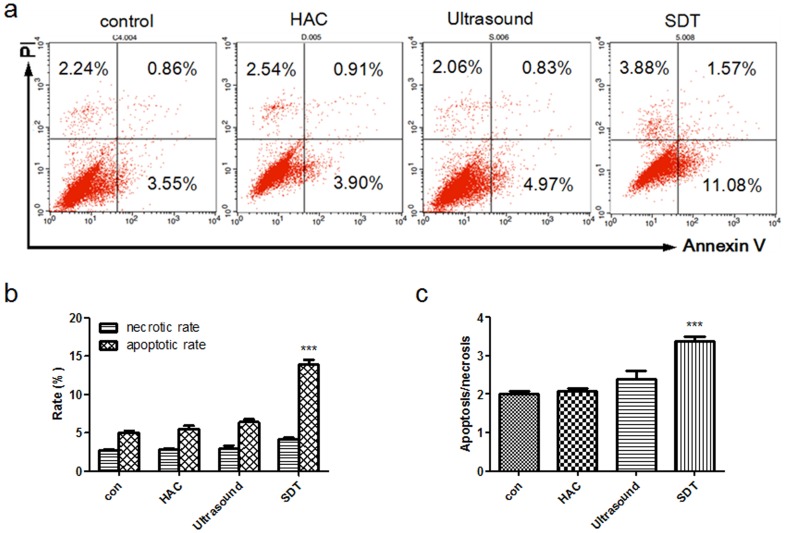
Apoptosis and necrosis of THP-1 macrophages determined by flow cytometry. (a) Apoptosis and necrosis rates of THP-1 macrophages of the control and HAC alone (5.0 μg/mL), ultrasound alone (5 minutes), SDT (5.0 μg/mL HAC plus 5 minutes ultrasound exposure) groups, measured using flow cytometry with double staining of Annexin V and PI. (b) The percentage of apoptotic and necrotic THP-1 macrophages determined by flow cytometry. (c) Apoptosis/necrosis ratio of THP-1 macrophages determined by flow cytometry. ***p<0.001 *vs.* control.

HAC-SDT induced both apoptosis and necrosis in THP-1 macrophages, and the apoptotic rate was higher than the necrotic rate with appropriate conditions, the maximum apoptosis/necrosis ratio was 3.39±0.20 in SDT group (Fig. 5c; p<0.01). The percentage of apoptotic cells increased significantly in SDT group (Fig. 5b; p<0.01). The early apoptotic ratio in the SDT group was much higher than that in the other groups (Fig. 5a). There was no obvious influence on cell apoptosis rates in HAC alone and ultrasound alone compared with control.

### Mitochondrial membrane potential

**Figure 6:**
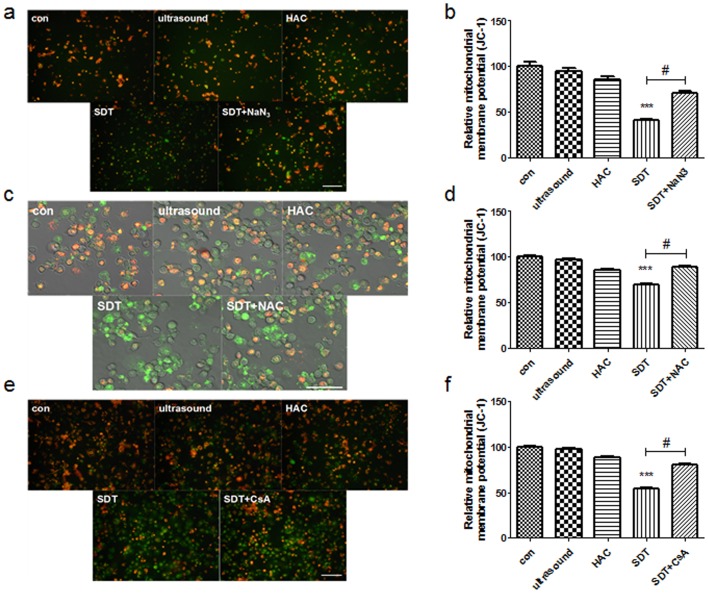
The Mitochondrial Membrane Potential determined by jc-1. (a) Fluorescent photomicrograph of THP-1 macrophages showing MMP. (b) The relative MMP level of THP-1 macrophages with NaN_3_. (c) Fluorescent photomicrograph of THP-1 macrophages showing MMP under laser confocal microscopy. (d) The relative MMP level of THP-1 macrophages with NAC. (e) Fluorescent photomicrograph of THP-1 macrophages showing MMP. (f) The relative MMP level of THP-1 macrophages with CsA. ***p<0.001 *vs.* control. #p< 0.05 *vs.* SDT. Scale bar  =  0.05 mm.

The green and red-orange fluorescence intensities of jc-1 were measured using the laser confocal microscopy and fluorescence microscope. The fluorescence properties of jc-1 in cells of the control and HAC alone, ultrasound alone, SDT and SDT+NaN_3_, SDT+NAC, SDT+CsA groups were monitored by the laser confocal microscopy and fluorescence microscope. Red-orange fluorescence was present in most cells of the control, HAC alone, ultrasound alone and SDT+NaN_3_, SDT+NAC, SDT+CsA groups, while green fluorescence was present in most of the SDT-treated cells (Fig. 6a, c, e). The relative MMP level of the ultrasound alone, HAC alone and SDT+NaN_3_, SDT+NAC, SDT+CsA groups were not different from that of control. However, the relative MMP levels of SDT were decreased significantly (Fig. 6b, d, f; p<0.01).

### ROS generation

**Figure 7:**
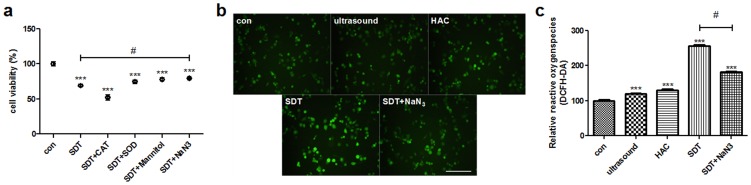
The ROS generation detection by DCFH-DA. (a) Effect of active oxygen scavengers on cell damage after SDT. To find out which one was the main inducer in apoptosis after SDT, several free radical scavengers such as 200 U/mL catalase (CAT, scavenger of hydrogen peroxide), 100 μg/mL superoxide dismutase (SOD, scavenger of superoxide anion radical), 100 mM mannitol (scavenger of hydroxyl radical) and 10 mM Sodium azide (NaN_3_, scavenger of singlet oxygen) were employed before treatment. The cell viability was checked by the CCK-8 assay. Ultrasonically induced cell damage were significantly reduced in the presence of NaN_3_ but not in the presence of CAT, SOD or mannitol, indicating singlet oxygen may be the main factor for cell damage. (b) Fluorescent photomicrograph of THP-1 macrophages stained by DCFH-DA showing intracellular ROS. (c) Intracellular ROS generation in THP-1 macrophages. ***p<0.001 *vs.* control. #p< 0.05 *vs.* SDT. Scale bar  =  0.1 mm.

ROS-induced damage was considered as the main mechanism underlying the effect of SDT, and the results confirmed ROS generation. The cells were pre-treated with several free radical scavengers such as catalase (CAT, hydrogen peroxide scavenger), superoxide dismutase (SOD, superoxide anion radical scavenger), mannitol (hydroxyl radical scavenger) and Sodium azide (NaN_3_, singlet oxygen scavenger). Cell viability was analyzed by the CCK-8 assay. The effects of CAT, SOD, mannitol and NaN_3_ after 5 min of insonation have been compared with SDT (Fig. 7a, #p< 0.05). When SOD or mannitol were incubated with cells, viability had improved trend. Only treatment with NaN3 reduced the HAC-SDT induced cell damage to a significant extent, indicating that singlet oxygen may be the main ROS inducing cell damage post-SDT.

The fluorescence intensity of DCF was measured by the fluorescence microscope. The green fluorescence of DCF was present in a few control cells, together with HAC alone and ultrasound alone, but most of the SDT-treated cells (Fig. 7b). ROS levels increased significantly in the SDT group (256.7%±13.45%) (Fig. 7c; p<0.01). The relative ROS level in the HAC alone and ultrasound alone increased not obviously compared with the control. Generation of reactive oxygen species decreased significantly in cells of the SDT group pretreated with the singlet oxygen scavenger, NaN_3_ compared with SDT group.

### Apoptosis initiated by SDT treatment

**Figure 8:**
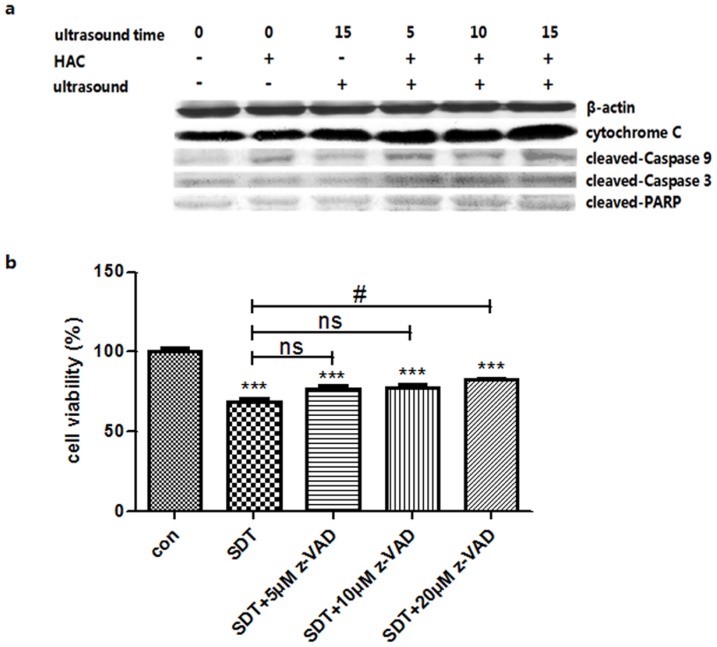
Protein levels changes were analyzed by western blot. (a) Protein levels of cytochrome C, cleaved-Caspase-9, cleaved-Caspase-3 and cleaved-PARP in the control and 5.0 μg/mL HAC alone, ultrasound alone, SDT (5.0 μg/mL HAC plus 5, 10 and 15 minutes ultrasound exposure) groups. β-actin was used as a loading control. (b) Effects of Caspase inhibitor z-VAD on cell viability of THP-1 macrophages. The cells were detected by CCK-8 assay after 6 h incubation following SDT with or without z-VAD pre-treatment. ***p<0.001 *vs.* control. #p< 0.05 *vs.* SDT.

The results showed that more obvious expressions of cytochrome C, Caspase-9 cleavage, Caspase-3 cleavage and PARP cleavage increased after SDT treatment when compared with control, HAC alone and ultrasound alone (Fig. 8a). The results showed the broad-spectrum Caspase inhibitor z-VAD rescued the cell death of THP-1 macrophages induced by SDT in a concentration dependent manner (Fig. 8b). These suggested SDT mediated apoptosis of THP-1 macrophages might be Caspase dependent.

## Discussion

As a photosensitizer, curcumin (excitation wavelength 425 nm, emission wavelength 530 nm) [22] has induced apoptosis of tumor cells [23]. Since hydroxy of curcumin is an unstable perssad, we acetylated the hydroxy of curcumin, named Hydroxyl acylated curcumin (excitation wavelength 407 nm, emission wavelength 476 nm), which was proved the photosensitizer (data not shown). However, its application was limited to superficial lesions because of light definite penetration. Low-intensity ultrasound has a much lower attenuation coefficient than light does in biological tissues [24], so in this study we used ultrasound instead of light to resolve the penetration depth.

Our previous studies have suggested that sonosensitizers combined with low-intensity ultrasound were effective for the removal of macrophages from unstable plaque. We demonstrated that SDT with emodin, curcumin and ALA could induce obvious macrophage apoptosis, which might relieve the further development of atherosclerosis [16], [19]–[20]. After the treatment of emodin-SDT, the cytoskeleton, significantly lost in its original features as the filaments dispersed and the cytoskeletal proteins aggregated [19]. While under the treatment of curcumin-SDT, mitochondrial membrane potential decreased and considerable ROS increased for the cytoskeleton [20]. Based on our earlier works, this study was to investigate the sonodynamic effects of Hydroxyl acetylated curcumin on macrophages *in vitro*.

Emodin, curcumin and ALA were targeted and accumulated in macrophages in our previous studies, while in this study we confirmed that the HAC also did in macrophages. There may be three possible reasons. Some studies indicated that curcumin up-regulated the expression of low-intensity lipoprotein receptor (LDL-R) protein in human lymphocytes, HEK-293 and mouse macrophages [25]–[30]. Liposoluble sensitizers likely enter cells through LDL-R, while both curcumin and Hydroxyl acetylated curcumin are defined as liposoluble sensitizers. Therefore, Hydroxyl acetylated curcumin maybe enters into macrophages through LDL-R. Moreover, low-intensity ultrasound could enhance cell membrane permeability [31]. And macrophages had the ability to phagocytose the HAC [32]. The uptake of HAC by macrophages showed its accumulation increased in accordance with its incubation time. CCK-8 assay measurement displayed that the cytotoxicity of HAC depended on its concentration and incubation time. These results indicated that the specification of safe drug concentration and incubation time were necessary for the effects of SDT.

The cell survival rate in the SDT group was much lower than that in the control, HAC alone and ultrasound alone under the same exposure conditions and drug concentration. Cell viability decreased gradually as the amount of ultrasound irradiation and drug concentration increased, which indicated that HAC-SDT could effectively kill macrophages *in vitro*. In this study, the temperature of the cell medium did not increase, which excludes any thermal effects (data not shown). However, CCK-8 assay did not indicate the mode of cell death.

In this study ultrasound exposure alone could induce cell apoptosis, which was highly enhanced with HAC. There was a synergistic relationship between HAC and ultrasound. The percentage of apoptotic and necrotic cells both increased, and the maximal apoptosis/necrosis ratio was 3.39 in SDT group. Clinically, apoptosis is preferred a better way to kill THP-1 macrophages over necrosis because it triggers less inflammation [33]–[34]. This study revealed that the apoptotic effect of ultrasonic irradiation on THP-1 macrophages was significantly enhanced with HAC. Early apoptosis was dominant over late apoptosis in SDT group, which was different from late apoptosis on the ALA-SDT treatment, and different from the apoptosis induced by the disruption of the cytoskeleton with curcumin-SDT [16], [20]. The cell apoptosis rate of the SDT group was much higher than that in other groups, while the HAC alone and ultrasound alone had little effect.

SDT induced apoptosis through the mitochondria-caspase signaling pathway in tumor [15], [35]–[36]. The loss of mitochondrial membrane potential was widely considered as the main pathway in SDT-induced apoptosis [37]–[39]. Our previous results confirmed that ALA mediated SDT significantly enhanced the THP-1 macrophages apoptosis by mitochondrial pathway [16]. In this study, the cells treated by ultrasonic irradiation displayed a decrease trend in mitochondrial membrane potential (MMP), and this decrease was significantly enhanced with HAC, while HAC alone had little effect.

The mechanism underlying SDT-induced apoptosis, which involves the generation of ROS, is controversial. ROS are a class of ubiquitous molecules such as hydroxyl radical, superoxide anion radical, hydrogen peroxide, and singlet oxygen, and have been implicated in many biological processes. ROS regulate critical steps in signal transduction cascades and many important cellular events. However, it is still controversial that which kind of ROS played a major role in inducing cellular apoptosis. In this study, the cells were treated with different free radical scavengers. The results indicated that singlet oxygen might be the main ROS inducing cell damage post-SDT. Therefore, we used the singlet oxygen scavenger NaN_3_ to confirm whether singlet oxygen was the main molecule involved in the effect of SDT. In the NaN_3_ group, the MMP increased significantly compared with SDT, which implied the singlet oxygen might be the main effector that caused mitochondrial damage and activated the mitochondrial signaling pathway. In the ROS inhibitor NAC group and MPTP inhibitor CsA group, the MMP had significantly inecreased compared with SDT, which indicated HAC-SDT mediated apoptosis may result from MMP reduction through targeting MPTP of mitochondrial. This was in agreement with our previous studies [16]. However it was different from other sonosensitizer-mediated SDTs through the cell membrane damage [19], [40]–[44].

Recent investigations have shown that increasing ROS production damaged mitochondria, which subsequently activated the signaling pathways that regulated cell apoptosis [45]–[47]. This study indicated that HAC alone and ultrasound alone led to similar ROS production compared with control, which also did not produce noticeable cell killing, growth inhibition or cell apoptosis. However, HAC-SDT induced much more ROS in THP-1 macrophages than control, HAC alone and ultrasound alone, which was consistent with previous studies of ALA-SDT in THP-1 macrophages [16]. The combination effect of the ultrasound and the HAC was responsible for the significant increase of cell damage and apoptosis, which suggested that HAC-SDT generated much more ROS in cells, corresponding well with the highest MMP loss and early apoptosis in the SDT group. The results implied the enhanced apoptosis of THP-1 macrophages by HAC-SDT was potentially related to the ROS-mediated mitochondrial function and activation of the mitochondria-caspase signaling pathway. We next attempted to determine which ROS played a pivotal role in the induction of apoptosis. Several free radical scavengers such as CAT, SOD, mannitol, and NaN_3_ were employed to study this effect. HAC-SDT induced cell damage was significantly reduced in the presence of NaN_3_ but not in the presence of CAT, SOD or mannitol. When SOD or mannitol were incubated with cells, viability had improved trend. However it had no significance in statistical analysis. While NaN3 had significance in statistical analysis. Coincubation with NaN3 significantly attenuated the generation of reactive oxygen species, indicating that HAC-SDT produced singlet oxygen in macrophages. This conclusion was consistent with that of Tang [48].

Apoptosis related proteins activation is the most critical events during the apoptosis. Many stimulating factors activate Caspase-9 proenzyme. The actived Caspase-9 in turn activates Caspase-3 that subsequently cleaves PARP, which leads to apoptosis [49]. The induction of apoptosis is a preferred mode of cell killing with less side effects and immune reactions [50]–[51]. Some studies suggested that apoptosis was induced in many tumor cells, and a mitochondria dependent pathway might be involved in SDT process [38], [48]. In this experiment, we confirmed that cytochrome C, cleaved-Caspase-9, cleaved-Caspase-3 and cleaved-PARP expression increased obviously after SDT treatment, which indicated that the Caspase dependent apoptosis pathway. In addition, when THP-1 macrophages were pre-treated with the Caspase spectrum inhibitor z-VAD, the cell viability in SDT was significantly increased when the concentration of z-VAD was up to 20 μM, indicating a Caspase dependent apoptosis pathway could be induced in THP-1 macrophages.

Several studies have reported that removal of macrophages from atherosclerotic plaques could attenuate inflammation and subsequent plaque progression [5], [33], [52]. HAC-SDT induced apoptosis of macrophages, indicating a useful and promising sonosensitizer for atherosclerosis. However, given that the role of macrophages in the atherosclerosis process is complicated, further investigations of HAC-SDT in animal models of atherosclerosis should be performed.
